# Supplementary material for: Replicable simulation of distal hot water premise plumbing using convectively-mixed pipe reactors
Source: PLoS One. 2020 Sep 16;15(9):e0238385. doi: 10.1371/journal.pone.0238385 (PMC7494094; doi:10.1371/journal.pone.0238385)
Supplement: S1 Fig — Incubator consisted of a box using a light bulb as a heat source. Temperatures were measured using an infrared thermometer temperature gun at various points along the pipe. (DOCX) [file pone.0238385.s001.docx]

**S1 Fig.** Temperature profile of clear PVC pipe filled with RO water after allowed to equilibrate with the room and incubator temperatures to undergo convective mixing at different angles. Incubator consisted of a box using a light bulb as a heat source. Temperatures were measured using an infrared thermometer temperature gun at various points along the pipe.
