# Supplementary material for: Replicable simulation of distal hot water premise plumbing using convectively-mixed pipe reactors
Source: PLoS One. 2020 Sep 16;15(9):e0238385. doi: 10.1371/journal.pone.0238385 (PMC7494094; doi:10.1371/journal.pone.0238385)
Supplement: S2 Fig — No significant correlation was found between total cell counts and either DO or total iron (Spearman rank correlation). (DOCX) [file pone.0238385.s002.docx]

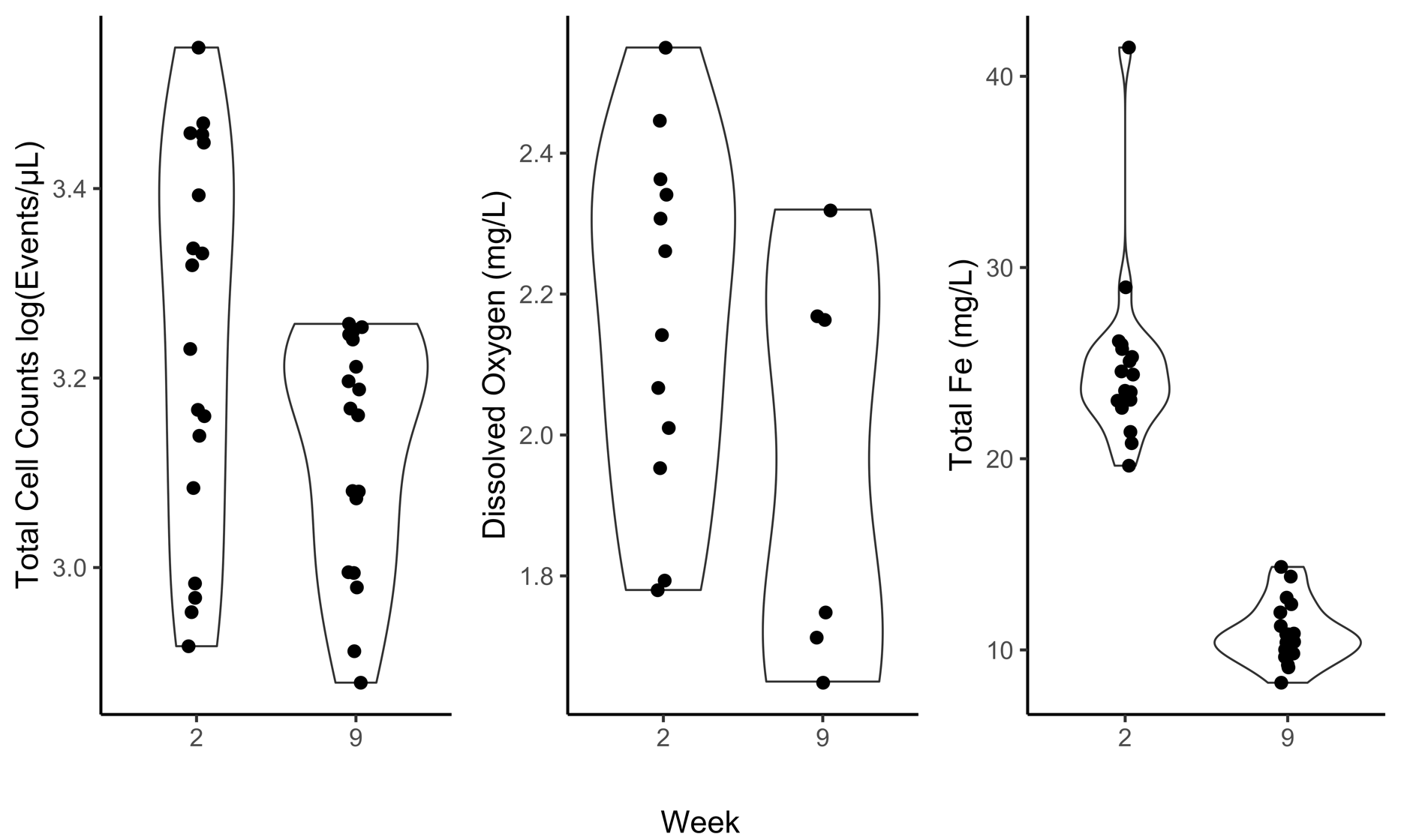


**S2 Fig.** Comparison of total cell counts, DO, and total iron in CMPR effluent in weeks 2 and 9. No significant correlation was found between total cell counts and either DO or total iron (Spearman rank correlation).
