# Supplementary material for: Replicable simulation of distal hot water premise plumbing using convectively-mixed pipe reactors
Source: PLoS One. 2020 Sep 16;15(9):e0238385. doi: 10.1371/journal.pone.0238385 (PMC7494094; doi:10.1371/journal.pone.0238385)
Supplement: S3 Fig — Naming convention: the first letter is a nominal indicator representing a set of 9 pipes representing a row of pipes in the CMPR housing unit, the second letter indicates pipe type (C = copper, F = PVC-iron, P = PVC), and the number represents the replicate number within the set of 9 pipes. All samples were collected during Week 10 with the exception of influent and seeded influent samples (Week 0). (DOCX) [file pone.0238385.s003.docx]

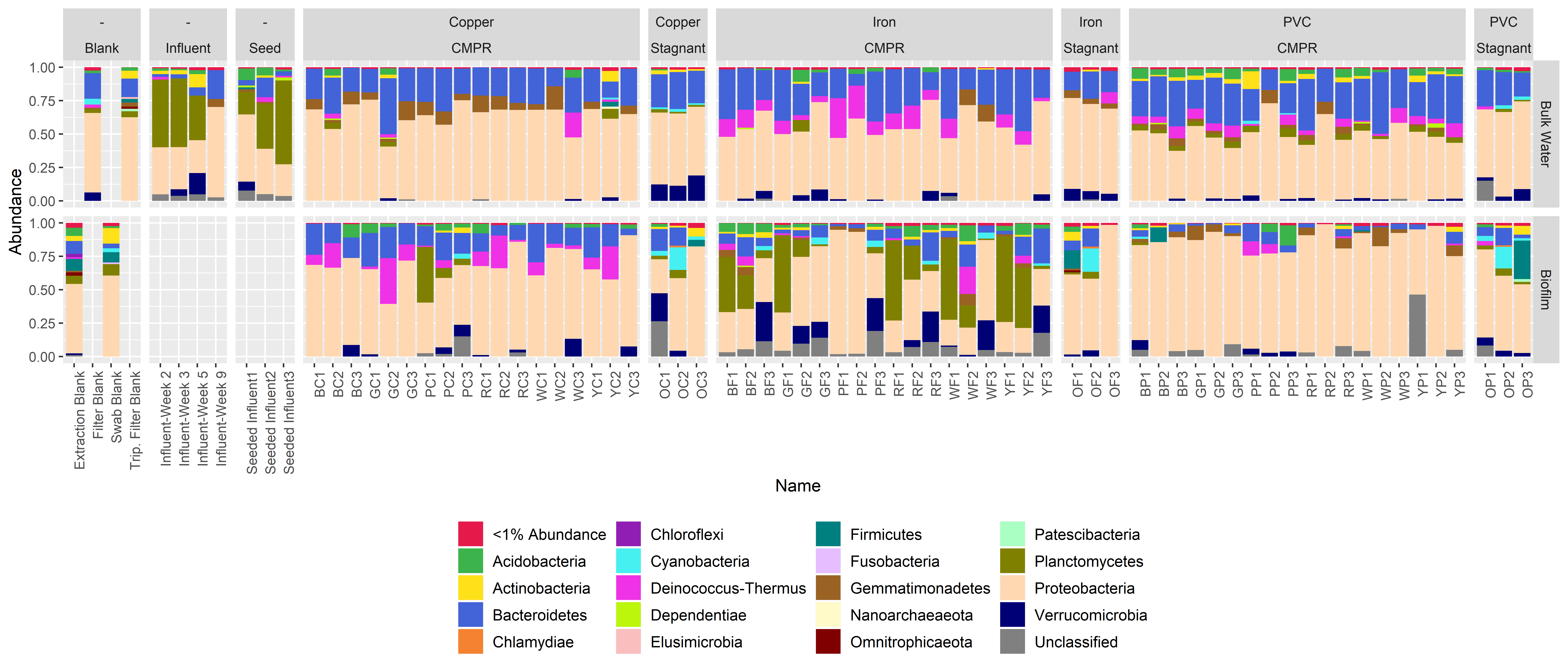


**S3 Fig.** Phylum level taxonomic microbial community profiles of each CMPR and each stagnant pipe organized by pipe type and location. Naming convention: the first letter is a nominal indicator representing a set of 9 pipes representing a row of pipes in the CMPR housing unit, the second letter indicates pipe type (C = copper, F = PVC-iron, P = PVC), and the number represents the replicate number within the set of 9 pipes. All samples were collected during Week 10 with the exception of influent and seeded influent samples (Week 0).
