# Supplementary material for: Replicable simulation of distal hot water premise plumbing using convectively-mixed pipe reactors
Source: PLoS One. 2020 Sep 16;15(9):e0238385. doi: 10.1371/journal.pone.0238385 (PMC7494094; doi:10.1371/journal.pone.0238385)
Supplement: S4 Fig — A single blank (filter blank) clusters near the majority of samples. This is assumed to be the result of cross-contamination during the preparation process given that all other blanks cluster separately. The microbial community compositions of blanks were found to be different than that of both bulk water and biofilm samples (Padonis < 0.01 for both unweighted and weighted metrics). (DOCX) [file pone.0238385.s004.docx]

**
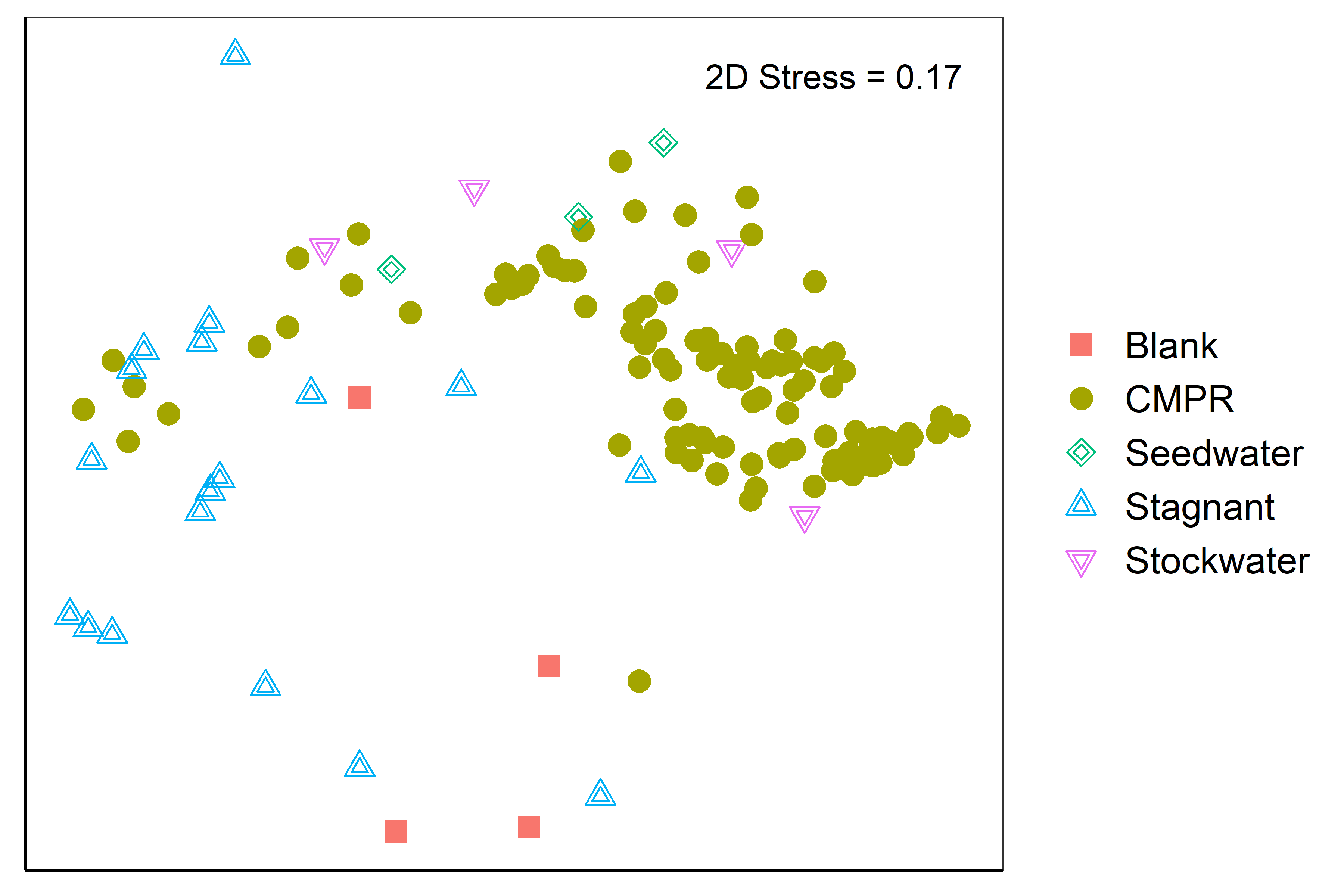
**

**S4 Fig.** Nonmetric multidimensional scaling (NMDS) plot, generated from Bray-Curtis dissimilarity matrix using the phyloseq package in R for 16S rRNA gene amplicon sequences rarefied to 1139 randomly selected reads to maintain all blanks and samples to compare taxonomic microbial community composition between blanks and samples. A single blank (filter blank) clusters near the majority of samples. This is assumed to be the result of cross-contamination during the preparation process given that all other blanks cluster separately. The microbial community compositions of blanks were found to be different than that of both bulk water and biofilm samples (P_adonis_ < 0.01 for both unweighted and weighted metrics).
