# Supplementary material for: Replicable simulation of distal hot water premise plumbing using convectively-mixed pipe reactors
Source: PLoS One. 2020 Sep 16;15(9):e0238385. doi: 10.1371/journal.pone.0238385 (PMC7494094; doi:10.1371/journal.pone.0238385)
Supplement: S5 Fig — Nonmetric multidimensional scaling (NMDS) plot, generated from Bray-Curtis dissimilarity matrix using the phyloseq package in R for 16S rRNA gene amplicon sequences, comparing CMPR (A) bulk water and (B) biofilm taxonomic microbial community composition. Bulk water and biofilm NMDS plots were generated independently. Pipe type was an important factor influencing the microbial community in both the bulk water Padonis = 0.001, R2 = 0.355, Pbetadis = 0.257) and biofilm (Padonis = 0.001, R2 = 0.309, Pbetadis = 0.001). (DOCX) [file pone.0238385.s005.docx]

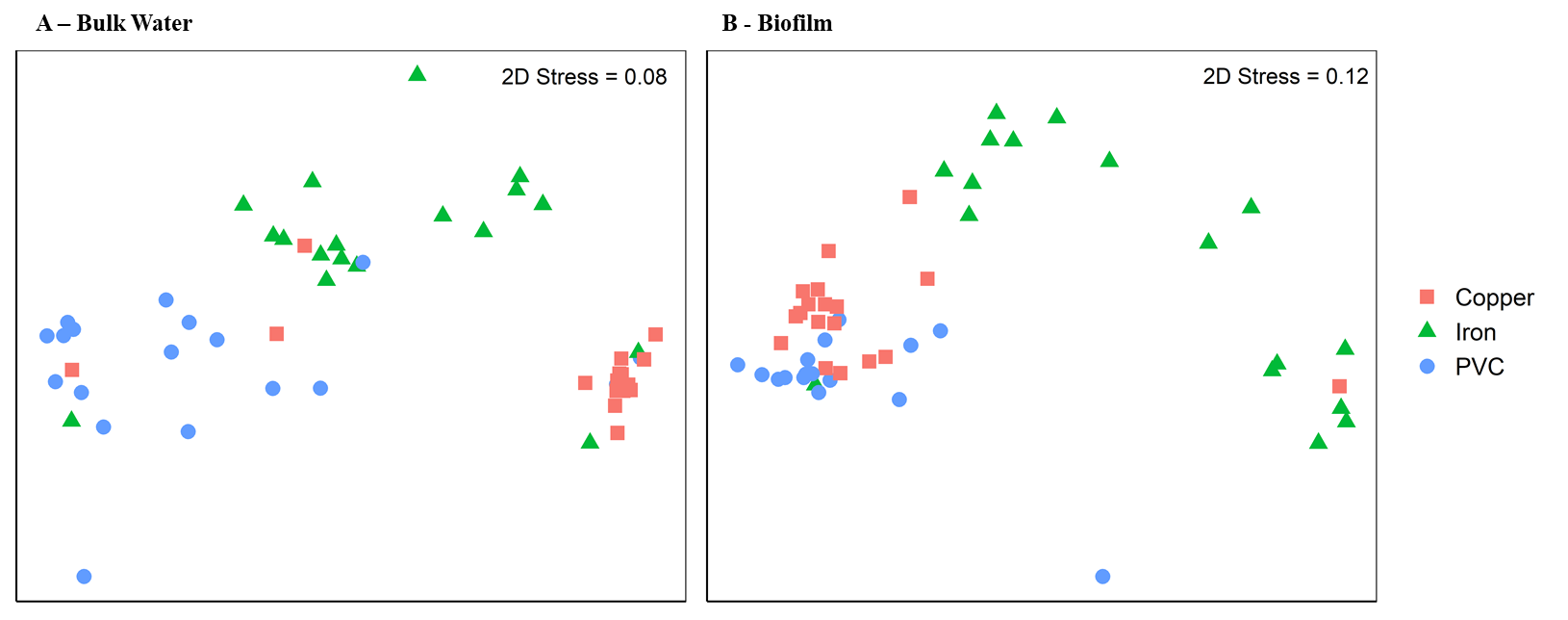


**S5 Fig.** Nonmetric multidimensional scaling (NMDS) plot, generated from Bray-Curtis dissimilarity matrix using the phyloseq package in R for 16S rRNA gene amplicon sequences, comparing CMPR (A) bulk water and (B) biofilm taxonomic microbial community composition. Bulk water and biofilm NMDS plots were generated independently. Pipe type was an important factor influencing the microbial community in both the bulk water P_adonis_=0.001, R^2^=0.355, P_betadis_= 0.257) and biofilm (P_adonis_=0.001, R^2^=0.309, P_betadis_= 0.001).
