# Supplementary material for: Replicable simulation of distal hot water premise plumbing using convectively-mixed pipe reactors
Source: PLoS One. 2020 Sep 16;15(9):e0238385. doi: 10.1371/journal.pone.0238385 (PMC7494094; doi:10.1371/journal.pone.0238385)
Supplement: S1 Table — (DOCX) [file pone.0238385.s006.docx]

**S1 Table.** Test data determining the maximum mixing velocity based on pipe angle using neutrally buoyant rhodamine dye.

|  | **Room Temperature End to Incubator** | | | | **Complete Cycle (End-to-End)** | | | |
| --- | --- | --- | --- | --- | --- | --- | --- | --- |
| **Pipe Angle** | **Distance [in]** | **Time [min]** | **Velocity [in/min]** | **ΔT [^o^C]** | **Distance [in]** | **Time [min]** | **Velocity [in/min]** | **ΔT [^o^C]** |
| **0^o^** | 20.0 | 17.70 | 1.1 | 5.4 | 72 | - | - | 7.6 |
| **5^o^** | 19.5 | 9.75 | 2.0 | 2.8 | 72 | 50 | 1.4 | 5.9 |
| **15^o^** | 19.0 | 5.50 | 3.5 | 1.5 | 72 | 19.5 | 3.7 | 2.0 |
| **30^o^** | 17.5 | 1.33 | 13.2 | 1.1 | 72 | 6.5 | 11.1 | 1.5 |
| **60^o^** | 18.0 | 0.60 | 30.0 | 1.2 | 72 | 3.5 | 20.6 | 0.5 |
| **90^o^** | 20.0 | 1.67 | 12.0 | 3.8 | 72 | - | - | 4.1 |
| **Room Temperature = 18.9 ^o^C** | |  |  |  |  |  |  |  |
| **Incubator Temperature = 33.1 ^o^C** | |  |  |  |  |  |  |  |
