# Supplementary material for: Replicable simulation of distal hot water premise plumbing using convectively-mixed pipe reactors
Source: PLoS One. 2020 Sep 16;15(9):e0238385. doi: 10.1371/journal.pone.0238385 (PMC7494094; doi:10.1371/journal.pone.0238385)
Supplement: S2 Table — (DOCX) [file pone.0238385.s007.docx]

| **S2 Table.** Physicochemical Characteristics of Influent Water | | | |  |
| --- | --- | --- | --- | --- |
|  | Mean |  | σ | n |
| **Temperature (°C)** | ~20* |  | - | - |
| **pH** | 7.51 | ± | 0.02 | 15 |
| **Total Chlorine (mg/L)** | 0.02 | ± | 0.01 | 15 |
| **TOC (mg/L)** | 0.86 | ± | 0.13 | 11 |
| **Soluble Cu (µg/L)** | 10.72 | ± | 0.54 | 10 |
| **Total Cu (µg/L)** | 11.87 | ± | 0.60 | 10 |
| **Soluble Fe (µg/L)** | 1.34 | ± | 1.52 | 10 |
| **Total Fe (µg/L)** | 1.16 | ± | 1.90 | 10 |
| *Water was acclimated to ambient room temperature before use. | | | | |
