# Supplementary material for: Replicable simulation of distal hot water premise plumbing using convectively-mixed pipe reactors
Source: PLoS One. 2020 Sep 16;15(9):e0238385. doi: 10.1371/journal.pone.0238385 (PMC7494094; doi:10.1371/journal.pone.0238385)
Supplement: S3 Table — (DOCX) [file pone.0238385.s008.docx]

**S3 Table.** Cost estimates for each type of premise plumbing simulation method.

| **Reactor Type** | **Component** | **Approx. Cost** | **Total Cost** |
| --- | --- | --- | --- |
| **Convective Mixing Pipe Reactors (CMPRs)** |  |  |  |
| Cost estimates via parts list during construction | Tank | $350 |  |
| *additional costs depend on | Tank Cover | $165 |  |
| no. of samples, pipe types, etc. | Water Heater | $265 |  |
|  | 2 x Pumps | $220 |  |
|  | Misc. Plumbing Supplies | $175 |  |
| Cost per Housing Unit |  |  | $1,175 |
| **Pilot-Scale Premise Plumbing Rigs** |  |  |  |
| cost estimates based on Rhoads et al., 2015 rig | 2 x Water Heater | $700 |  |
| *Cost can vary depending on rig | 8 x Solenoids | $1,200 |  |
|  | 2 x Chrontrols | $800 |  |
|  | 3x GAC filtration | $120 |  |
|  | 16 x Flow Rate Rotameters | $800 |  |
|  | Misc. Plumbing Supplies | $500 |  |
| Cost Per Rig |  |  | $4,120 |
| **CDC Biofilm Reactor** |  |  |  |
| <https://biofilms.biz/products/biofilm-reactors/cdc-biofilm-reactor/> | Reactor System | $990 |  |
|  | Digital Stir/Hot Plate | $1,490 |  |
| Cost Per Reactor |  |  | $2,480 |
| **Simulated Glass Water Heaters (SGWHs)** |  |  |  |
|  | 120 mL glass bottle | $354/120 bottles |  |
| Cost per SGWH |  |  | $3 |
