# Supplementary material for: Replicable simulation of distal hot water premise plumbing using convectively-mixed pipe reactors
Source: PLoS One. 2020 Sep 16;15(9):e0238385. doi: 10.1371/journal.pone.0238385 (PMC7494094; doi:10.1371/journal.pone.0238385)
Supplement: S2 File — (DOCX) [file pone.0238385.s010.docx]

**Influence of Drawing Lubricant on**

**Copper Release from**

**Copper Pipes During NSF 61 Testing**

**Roger Arnold**

**Marc Edwards**

**Virginia Tech**

**Draft Final Report**

**August 20, 2010**

**Variability in Copper Release for Manufactured Pipes**

Each manufacturer in the U.S. must regularly certify copper pipes using NSF/ANSI Standard 61 testing. Prior research has demonstrated considerable variability (≈ 200-300%) in copper release during testing, even for copper pipes produced from the same batch. This recently created problems for one manufacturer, because a pipe selected for testing failed to meet the NSF criteria. It is hypothetically possible that the variability is due to the NSF test, to slight differences between pipes arising during manufacture, or from a combination of these factors.

To isolate whether the variability is due to the pipes or due to testing, four 4-foot sections of copper pipe deemed representative of the process, were exposed to synthetic water according to NSF Standard 61 (Table 1). Testing was conducted by Dr. Marc Edwards "head to head," putting the exact same water into each pipe, to essentially eliminate substantive variability from the test protocol or the test water. Pipes were emptied and refilled three times per week, and samples were analyzed using inductively coupled plasma mass spectrometry (ICP-MS).

Results demonstrated highly reproducible differences between the pipes when exposed to the exact same NSF test water. Specifically, pipes exhibiting “Group 1” behavior started with very low copper leaching but rose to sustained high levels (Figure 1). Pipes exhibiting “Group 2” released lower levels of copper for the later stages of testing. At the time NSF samples are collected to determine if the product passes testing (about 3 weeks), Group 2 samples contained 2-3 times more copper than did Group 1 samples. Thus, it became important to understand the cause for the difference in copper release observed amongst the tubes.

**Figure 1.** Copper Pipes exposed to NSF Extraction Water

An important clue was detected from analysis of trace metals present in the water of the "Group 1" samples. Specifically, traces of W, Ni, Co, and P were in the water, and these elements were suspected to be part of the drawing plug. An investigation was conducted by the manufacturer that focused on issues that would alter the interaction of the drawing plug with the copper tube surface.

**Pipe Lubrication**

A detailed evaluation of the manufacturing process indicated that there were dramatic differences in tube lubrication at various parts of a run. Specifically, lubricant was not being added for some of the passes. It was hypothesized that for tubes produced without lubricant, there was increased friction or interaction between the drawing plug and tube, which left a trace element "fingerprint" from the drawing plug on the copper surface, and somehow increased the propensity of the tube to release copper in NSF testing.

To test this hypothesis under controlled conditions with a larger sample size, a simulated 3-week NSF/ANSI Standard 61 test was performed to evaluate whether the use of a lubricant during extrusion could affect copper pipe certification. Ten tubes were manufacturered without application of lubricant and ten tubes were manufactured with lubricant. As before, all pipes were filled with the NSF test water (Table 1) with water changes three times per week.

**Copper Release Results**

The batch of samples produced without lubricant started out with much lower levels of copper release than did the batch of sample produced with lubricant (Figure 2). However, whereas the copper release remained relatively constant for the case of tubes produced with lubricant (+/- 25%), the copper release increased by more than 700% for tubes produced without lubricant over the three weeks of the study. Copper release from un-lubricated pipes was significantly lower at the first-flush and significantly higher after the second week (Table 2). Copper release was also much more variable for tubes produced without lubricant (Figure 3), with a standard deviation amongst the 10 replicates that was 4X higher than for tubes produced with lubricant. The higher variance increases the chance that an unrepresentative pipe sample will be chosen for certification testing, and that the tube would fail to meet NSF standards.

**Figure 2.** The Effect of Lubricant on Copper Leaching Behavior

**Table 2**. Statistical Summary of Copper Release

**Figure 3.** Effect of Lubricant on Copper Leaching Variability

Additionally, in both experiments, a “fingerprint” of W, Ni, Co, and P was detected in the first-flush samples of pipes extruded without a lubricant (Table 3). X-ray fluorescence spectrometry of the mandrel used to extrude copper pipes confirmed a surface composition of W, Co, and Ni. We speculate that the presence of trace amounts of these metals on the interior surface of the pipe may have provided cathodic protection to the copper during the first sampling event when copper leaching was very low, but that copper release later rose from the un-lubricated pipes due to detrimental scratches or micro-imperfections in the tube arising from the higher friction. Scanning Electron Microscope (SEM) images of the internal copper pipe surface prior to exposure to the water, showed no conclusive visual differences on the surface of tubes produced with and without lubricant. However, the surface scan of the new copper pipes without lubricant did detect significantly more W and P (Table 4).

**Table 3.** Signature Elements Present in First-flush Water Samples

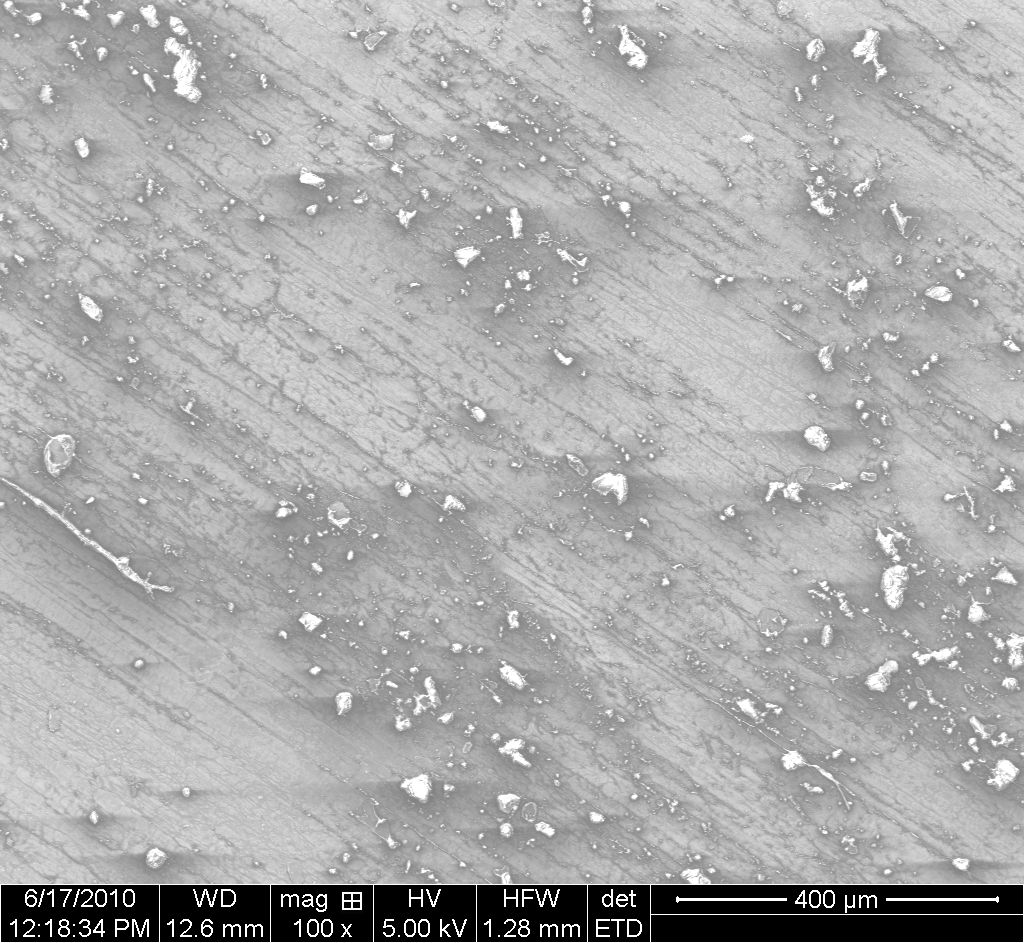

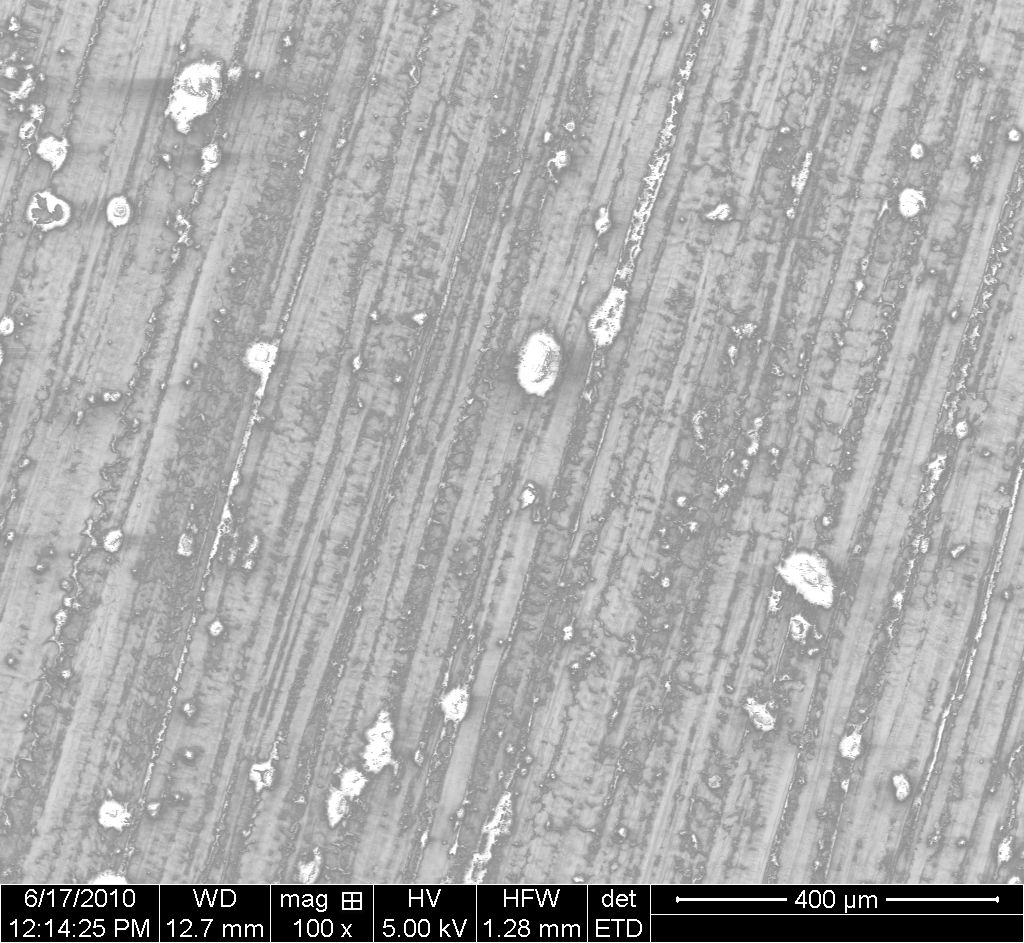

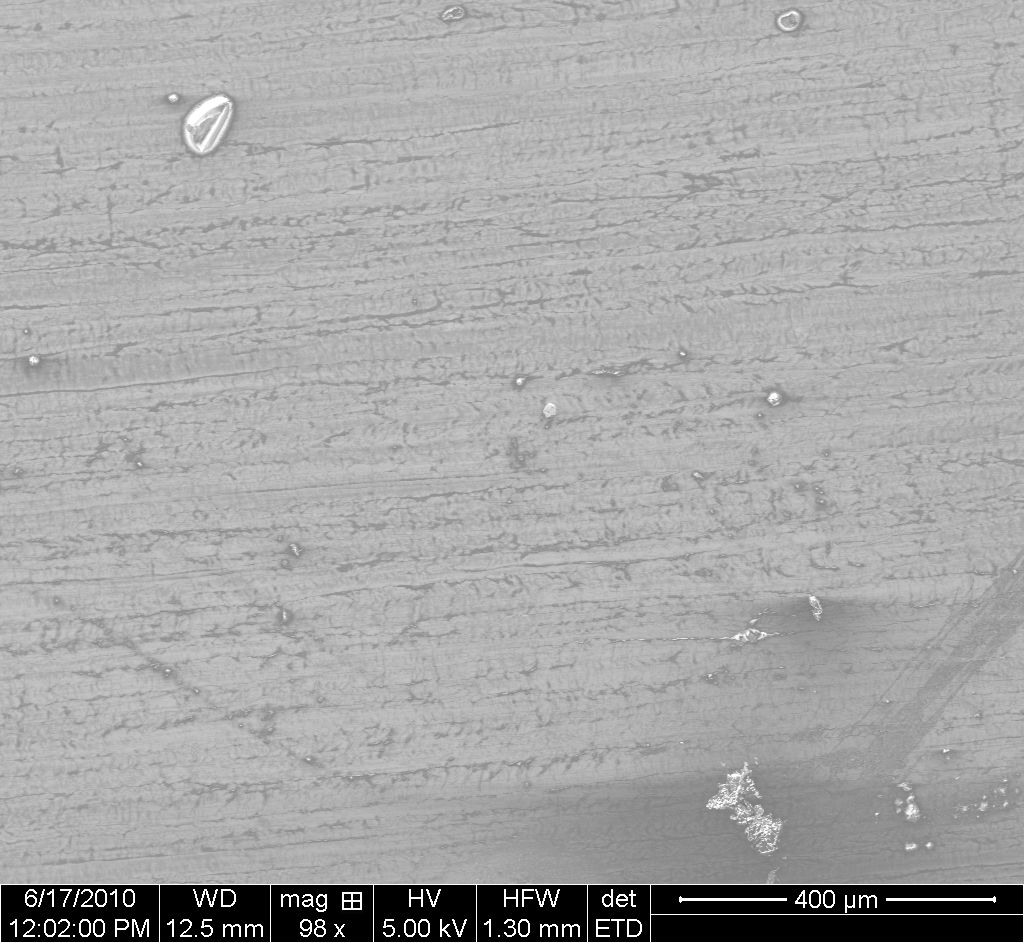

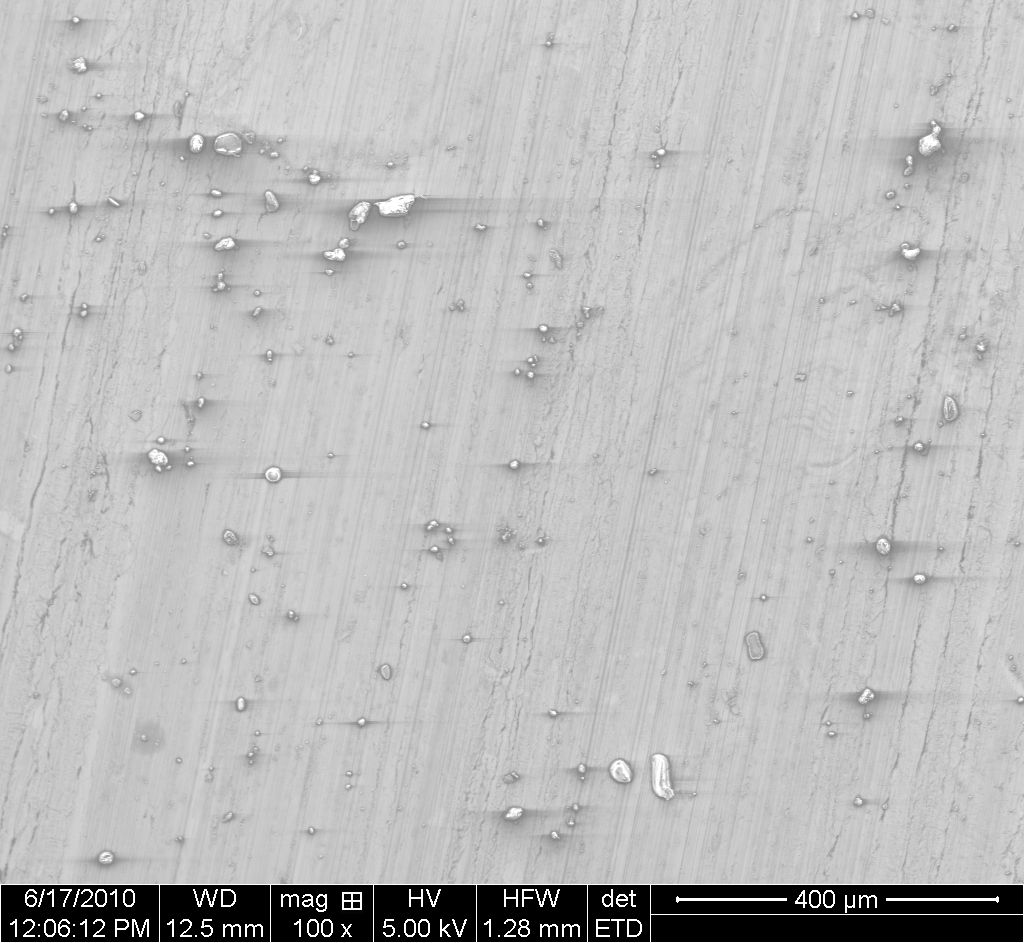


a.) SEM images of copper pipe manufactured with lubricant

b.) SEM images of copper pipe manufactured without lubricant

**Figure 4.** SEM Images of Copper Pipe Samples Prior to Exposure to Water

**Table 4.** Elements Detected by Surface Scan on Pipe Surface

**Conclusions**

- There can be a high variability of copper release (up to 600%) from manufactured tubes, even within the same batch of samples, that is due to variability in the manufacturing process.
- For one set of tubes, application of lubricant increased the initial copper in the water, but decreased the copper release to the water over the long-term. The use of lubricant also dramatically reduced the variability in copper release from tube to tube.
- The absence of a lubricant during manufacturing can increase the concentration of trace metals on the pipe surface, most likely due to increased friction between the tube and the mandrel. This somehow translates to a dramatically increased likelihood of failure during NSF Standard 61 certification.
